# An in-depth examination of variations in cerebral venous sinuses and the occurrence of sinovenous thrombosis

**DOI:** 10.1186/s12880-026-02294-3

**Published:** 2026-03-23

**Authors:** Koray Bingol, Hatice Kubra Ozdemir

**Affiliations:** 1 Department of Anatomy, Erzincan Binali Yıldırım University Mengücek Gazi Training and Research Hospital, Erzincan, Türkiye; 2 Department of Radiology, Etlik City Hospital, Ankara, Türkiye

**Keywords:** Pediatric, Cerebral sinovenous thrombosis, Cerebral venous anatomical variations, MR venography

## Abstract

**Background:**

Pediatric cerebral sinovenous thrombosis (CSVT) is an uncommon yet serious cause of stroke in children. Although cerebral venous anatomical variations (AVs) are frequently encountered on MR venography (MRV), their potential contribution to CSVT remains unclear. Only one prior study has explored the relationship between AVs and CSVT, highlighting the need for larger population-based investigations. This study aimed to examine the prevalence of AVs in pediatric CSVT and non-CSVT populations and to assess their potential association with thrombosis and CSVT-related complications.

**Methods:**

This retrospective study included 124 children who underwent MRV between January 2020 and November 2025. Sixty-two patients diagnosed with CSVT were age- and sex-matched with 62 controls. MRV examinations were evaluated for the presence and subtype of AVs. Clinical presentation, complications, and demographic characteristics were recorded.

**Results:**

AV prevalence did not differ significantly between CSVT and control groups (46.7% vs. 50%, *p* = 0.67). The most common variation in both groups was left hypoplastic transverse sinus. AV rates were also similar between complicated and uncomplicated CSVT cases (44.4% vs. 47.1%, *p* = 0.92). Headache was the predominant presenting symptom (70.1%). Complications—including papilledema, elevated intracranial pressure, and venous infarction—were more common in younger patients (*p* = 0.01).

**Conclusions:**

AVs are common findings on pediatric MRV; however, their presence does not appear to influence CSVT development or complication risk. This study represents the largest cohort evaluating AVs in pediatric CSVT and contributes valuable evidence suggesting that AVs are incidental rather than pathogenic in this context.

## Introduction

Pediatric cerebral sinovenous thrombosis (CSVT) is an increasingly recognized form of stroke in children, accounting for approximately 0.5–3% of all pediatric strokes. Its annual incidence in the general pediatric population is estimated at 0.4–0.7 per 100.000 children. The age distribution follows a U-shaped pattern, with the highest frequency observed during the neonatal and infant periods—approximately five times higher than in other age groups [1].

In contrast to arterial growth, cerebral venous development is a largely passive process driven by hemodynamic and circulatory factors activated in response to local oxygen concentration, among other variables. Consequently, venous architecture exhibits significant variability among people [2].

Doppler ultrasound, computed tomography (CT), CT venography (CTV), magnetic resonance imaging (MRI), and magnetic resonance venography (MRV) have complimentary functions in assessing pediatric patients with venous abnormalities. The choice of modality and technique is contingent upon the clinical environment and the condition in question. The objective of pediatric cerebral venous imaging protocols is to efficiently resolve the clinical issue while minimizing factors such as delayed imaging due to imaging unit availability, exposure to procedural sedation, artifacts from patient motion, utilization of intravenous contrast material, and exposure to ionizing radiation. Magnetic Resonance Imaging (MRI) is the predominant technique employed for visualizing pediatric cerebral venous abnormalities. In addition, many pediatricians prefer MRV as the imaging method as it allows to avoid ionising radiation. MRV is crucial for examining the anatomy of the cerebral venous system and its variations to identify any pathology and prevent misinterpretation of typical variations [2, 3].

The frequency of CSVT, treatment approaches, and interventional differences, as well as the causes of mortality and morbidity, have been extensively addressed in various studies. However, only one study has investigated the potential relationship between venous anatomical variations (AV)—a commonly encountered phenomenon—and the presence of thrombosis, and it was conducted on a relatively limited population [4].

The primary objective of the present study is to examine the potential impact of AV on thrombosis development and to identify differences in the prevalence of AV between children with and without CSVT.

## Materials and methods

### Ethical aspects

This study obtained ethical approval from the Clinical Research Ethics Committee at Erzincan Binali Yıldırım University (Protocol number: EBYU-KAEK-2024–11/01/Date: 22 August 2024). Due to the study’s retrospective nature, informed consent was waived.

### Study design

**Fig. 1:**
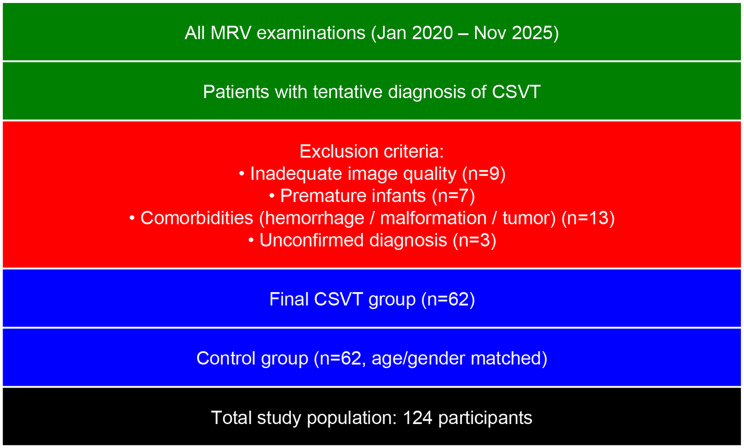
Flowchart of patient selection and study population

The main objective of this study was to examine the potential effect of AVs on thrombosis development and to reveal the differences in AV prevalence in children with and without SSVT. For this purpose, all patients who underwent MRV with a preliminary diagnosis of SSVT between January 2020 and November 2025 were retrospectively analyzed. Patients were excluded if their MRV images lacked adequate quality for assessment, if they were premature infants, if they had any comorbid conditions (such as intracranial hemorrhage, vascular malformation, or tumors), or if their CSVT diagnosis was not corroborated by clinical data or subsequent imaging. In addition, patients in whom a reliable distinction could not be made between chronic venous sinus thrombosis and congenital hypoplasia or aplasia were excluded from the study to prevent misclassification. In this setting, 62 patients with cerebral venous sinus thrombosis were found. Sixty-two normal participants, matched by age and gender, were randomly selected to constitute a control group. The diagnoses of the control group patients included in the study are detailed in Table 1, and the study was conducted accordingly. Patients with findings including hemorrhage leading to cerebral edema that made venous structure assessment difficult or impossible were not included in the study. The study population consisted of 124 patients (Fig. 1).

**Table 1:** Final diagnosis of the control group

Final Diagnosis	n (%)
No follow-up/unknown	11 (26.1%)
Cerebral infections	14 (30.9%)
Migrane	7 (16.6;%)
Tension-type headache	6 (14.2%)
Visual impairment	3 (7.1%)
Due to medications-side effects	1 (2.3%)
Total	42 (100%)

The diagnosis of CSVT was validated using clinical follow-up, imaging studies, and radiological assessments of the cases.

### MRV protocol

Magnetic resonance imaging was conducted using a superconducting 1.5-T MR system (Siemens Magnetom Area, Germany) and a conventional head coil. Three-dimensional MR venography was conducted in the coronal plane with the subsequent parameters: TE-50, TR-500, FOV 230–250 mm, slice thickness 2 mm, matrix 240 × 256, flip angle 60 degrees. Supplementary protocols included T2-weighted imaging in coronal and axial FLAIR sequences. A slice thickness of 1.2 mm was obtained with contiguous sections utilizing a matrix size of 256 × 256 and a notional field of view (FOV) of 25 cm, with a repetition time (TR) of 25 milliseconds, an echo time (TE) of 7.15 milliseconds, and a flip angle of 25 degrees. We incorporated contrast-enhanced T1-weighted imaging and, when required, contrast-enhanced MR venography images.

### Evaluation of the images

All images were evaluated by each investigator. The presence of AV was determined by consensus among the investigators. Data regarding age, sex, presence of AV, type of AV, presence of any complications, and predominant complaint at presentation were recorded.

### Statistical analysis

Data were analyzed using the Package for Social Sciences (SPSS) 25 for Windows (IBM SPSS Inc., Chicago, IL, USA). Normal distribution of the data was evaluated using the Kolmogorov–Smirnov test. Numerical variables with normal distribution are shown as mean ± standard deviation. The variables not with normal distribution are shown as median, minimum–maximum values. Categorical variables are shown as numbers and percentages. For the comparison of numerical variables between CSVT and control subgroups, Mann–Whitney U test and Student’s t-test were used. For the comparison of categorical variables, the Fisher’s exact or Chi-squared tests were applied.

A two-tailed value of *p* < 0.05 was considered statistically significant.

## Results

The study population comprised 124 participants, including 62 children from each group (CSVT and control). The mean age was 6.2 ± 1.3 years in the CSVT group and 5.9 ± 1.1 years in the control group, with no statistically significant difference between the two groups (*p* = 0.9). The CSVT group comprised 37 girls (59.6%) and 25 boys (40.3%), while the control group consisted of 35 girls (56.4%) and 27 boys (43.5%). No significant difference in gender was observed between the groups (*p* = 0.78).

Out of the MRV examinations, 23 (18.5%) were conducted with IVCM injection, whereas the rest were non-contrast studies. CSVT was identified in 8 out of 23 examinations (34.7%), whereas the remaining cases were classified within the control group.

Upon reviewing the preliminary diagnoses and examination rationales of the cases, the predominant indication was severe headache (87/124, 70.1%). The comprehensive distribution of indications is illustrated in Table 2.

**Table 2:** Reasons for performing an MRV examination

Indication	Total n (%)	CSVT n (%)	Control n (%)
Severe headache	87 (70.1%)	45 (67.2%)	42 (73.7%)
Altered level of consciousness	12 (9.7%)	6 (9.0%)	6 (10.5%)
Visual disturbance	10 (8.1%)	5 (7.5%)	5 (8.8%)
Nausea and vomiting	9 (7.3%)	5 (7.5%)	4 (7.0%)
Papilledema	6 (4.8%)	6 (9.0%)	0 (0%)

The AV ratio showed no significant difference between the CSVT and control groups (29–46.7% vs. 31–50%, respectively; *p* = 0.67). The predominant anatomical variant in all groups was the left hypoplastic transverse sinus (CSVT 8 - 27.5%, control 9–29%). While duplication/fenestration/septation variation was more prevalent in the control group, and disconnected superficial and deep systems variation was more frequent in the CSVT group, the limited patient sample precluded a statistically significant analysis. Table 3 provides a comprehensive distribution of all AV categorized by groups.

**Table 3:** Demographic data and distribution of AVs by subgroups

	CSVT	Control
Age	6.2 ± 1.3	5.9 ± 1.1
Gender (M/F) (n, %)	25, 40.3%-37, 59.6%	27, 43.5%-35, 56.4%
**Anatomic Variations**		
**Hypoplasia/aplasia**	**15 (51.7%)**	**17 (54.8%)**
Hypoplastic transvers sinus-Left	8 (27.5%)	9 (29%)
Hypoplastic transvers sinus-Right	4 (13.7%)	6 (19.3%)
Hypoplastic transvers sinus-Bilateral	2 (6.8%)	1 (3.2%)
Hypoplastic sigmoid sinus- Right	1 (3.4%)	1 (3.2%)
**Persistent fetal structures**	**10 (34.4%)**	**10 (32.2%)**
Left occipital sinus	3 (10.3%)	4 (12.9%)
Right occipital sinus	5 (17.2%)	5 (16.1%)
Bilateral occipital sinuses	2 (6.8%)	1 (3.2%)
**Duplication/fenestration/septation**	**2 (6.8%)**	**4 (12.9%)**
Duplication/fenestration of superior sagittal sinus	1 (3.4%)	2 (6.4%)
Double-channel straight sinus	1 (3.4%)	2 (6.4%)
**Disconnected superficial and deep systems**	**2 (6.8%)**	**0 (0%)**
**TOTAL**	29 (46.7%)	31 (50%)

CSVT: Pediatric cerebral sinovenous thrombosis

As shown in Table 3, hypoplastic transverse sinus was the most frequently observed condition in patients. Less frequently, occipital sinus variants and duplication/fenestration were observed, more often in the superior sagittal sinus.

**Fig. 2:**
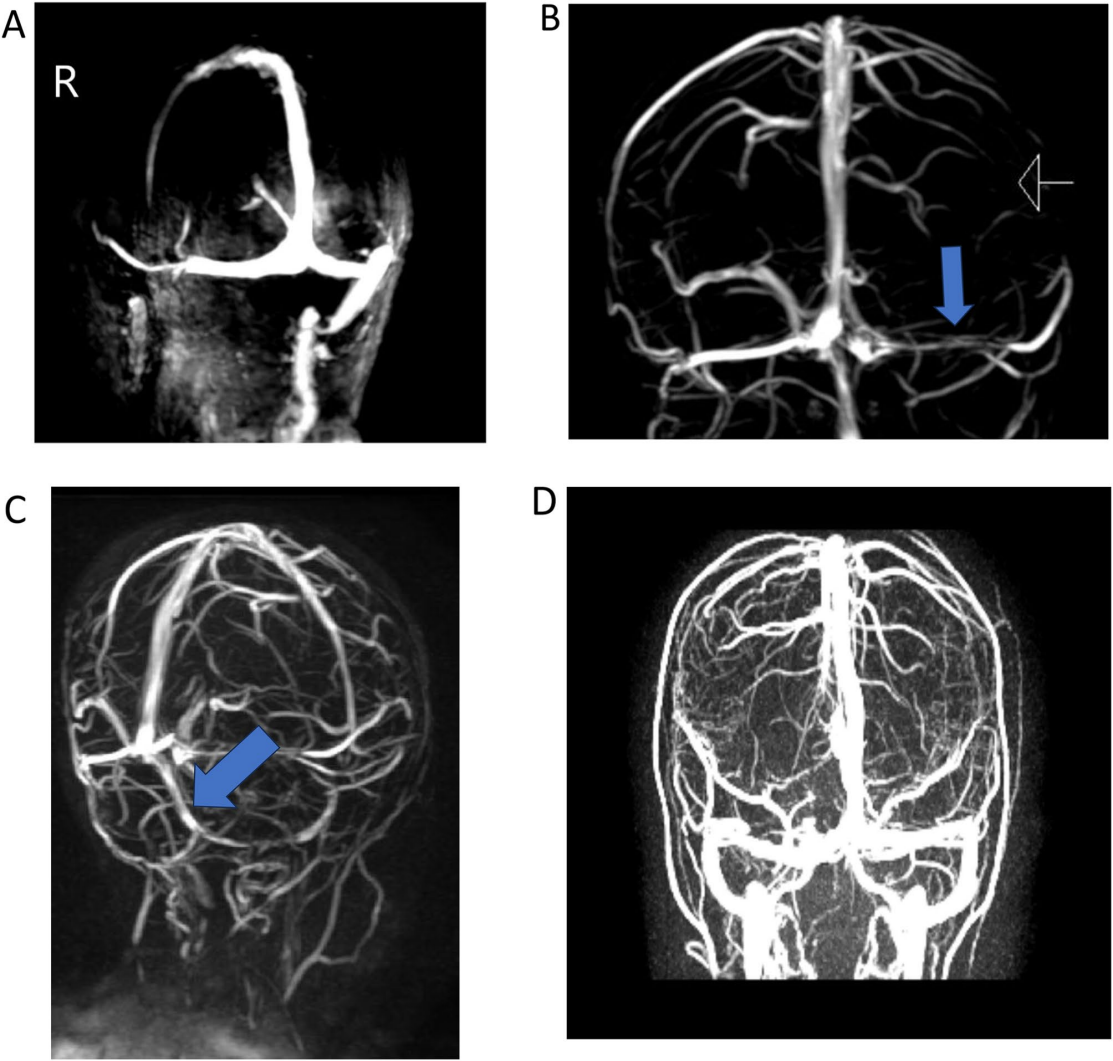
(**A**) Hipoplastic right sigmoid sinüs. (**B**) Hipoplastic left transverse sinüs. (**C**) Persistent occipital sinus (**D**) Normal MR venograhy image

Some of the detected AVs are shown in Figs. 1 and 2.

Upon analysis of CSVT cases for complications, 9 cases (9/62, 14.5%) exhibited complications, including papilledema and elevated intracranial pressure identified in 6 instances, and cerebral venous infarction noted in 3 cases. Median age is statistically significant in complicated cases (0 vs. 6, *p* = 0.01). AV detection rates were comparable for the complicated and uncomplicated CSVT groups (4–44.4%, 25–47.1%, respectively, *p* = 0.92). The predominant AV in both groups was the existence of a hypoplastic left transverse sinus. Table 4 delineates the distribution of AV within the CSVT subgroups based on the occurrence of complications.

**Table 4:** Distribution of the demographic data and AVs by complicated and noncomplicated CSVT cases

	CSVT	
	Complicated (n = 9)	Noncomplicated (n = 53)
Age (median, min.-max)	0 (0–3)	6 (0–17)
Gender (M/F) (n, %)	4–44.4%, 5–55.5%	12–60%, 8–40%
**Anatomic Variations**		
**Hypoplasia/aplasia**	**3 (33.3%)**	**12 (60%)**
Hypoplastic transvers sinus-Left	2 (22.2%)	6 (30%)
Hypoplastic transvers sinus-Right	1 (11.1%)	3 (15%)
Hypoplastic transvers sinus-Bilateral	0	2 (10%)
Hypoplastic sigmoid sinus- Bilateral	0	1 (5%)
**Persistent fetal structures**	**1 (11.1%)**	**9 (45%)**
Left occipital sinus	0	3 (15%)
Right occipital sinus	1 (11.1%)	4 (20%)
Bilateral occipital sinuses	0	2 (10%)
**Duplication/fenestration/septation**	**0**	**2 (10%)**
Duplication/fenestration of superior sagittal sinus	0	1 (5%)
Double-channel straight sinus	0	1 (5%)
**Disconnected superficial and deep systems**	**0**	**2 (10%)**
**TOTAL**	4 (44.4%)	25 (47.1%)

## Discussion

SSVT is a rare but serious type of stroke that can occur from the prenatal period to puberty. It is more common in the neonatal period, and the incidence of complications is inversely proportional to age [5]. The main objectives of this study were to examine the frequency of AVs in SSVT cases, to compare them with normal cases, and to evaluate the possible effect of AVs on outcomes related to the onset of SSVT. In our study, no significant relationship was shown between detected AVs and SSVT or its complications. However, it was found that the median age of patients who developed complications was lower.

The initial signs of pediatric cerebral venous thrombosis (CSVT) vary by age group. Symptoms in the younger age group are headache in approximately 85–90% of cases, vomiting in approximately 70%, and visual disturbances such as blurred or double vision due to papilledema in approximately 40%, all associated with increased intracranial pressure and localized neurological disorders. In older pediatric patients, approximately one-third may experience acute seizures and focal motor disturbances, including hemiparesis. In infants, apnea or respiratory distress is often observed as the initial symptom. It is emphasized that in age-related differences, newborns with CSVT usually present with nonspecific symptoms such as seizures, malfeeding, and restlessness [6].

Upon examining the symptomatology distribution in our study cohort, headache emerges as the predominant reason for admission, aligning with existing literature. Likewise, headache is accompanied with altered consciousness, visual disturbances, nasal congestion, and vomiting, listed in order of prevalence. The consistent symptomatology of the study sample, in conjunction with existing literature, bolsters the credibility of the results presented.

MRV is essential in diagnosing pediatric CSVT, providing a noninvasive method to verify venous sinus occlusion without subjecting children to ionizing radiation. MRV is considered the preferred imaging technique for children with suspected CSVT, as it directly visualizes thrombus (intraluminal filling defects and absence of flow) and can concurrently identify any venous infarction or underlying structural abnormalities. In the pediatric demographic, conventional non-contrast MRV methodologies (e.g., time-of-flight MRV) are constrained by flow-related aberrations and developmental venous anomalies (such as sluggish flow or hypoplastic sinuses) that may resemble thrombosis, potentially resulting in false-positive results. Thus, contrast-enhanced MRV (CE-MRV) is favored for better diagnostic precision: gadolinium enhancement generates a luminous intravascular “lumenogram” irrespective of flow dynamics, aiding in the differentiation of genuine occlusions from sluggish flow or anatomical variations. Recent investigations highlight the efficacy of CE-MRV, demonstrating high sensitivity and specificity (about 83 and 100%, respectively) in diagnosing CSVT, greatly surpassing non-contrast MRV sequences and equaling the diagnostic yield of CT venography. Consequently, MRV – especially with contrast enhancement – is essential in the pediatric CSVT evaluation, optimizing the identification of venous thromboses (even those in minor cortical veins) while eliminating radiation exposure and minimizing diagnostic uncertainty linked to non-contrast methods [2, 7].

IVCM was utilized in around 18% of the cases included in our study. This may be regarded as a limiting factor due to the significant diagnostic capability of contrast-enhanced MRV; yet, pathology was identified in merely 34.7% of instances utilizing IVCM, with the rest seeming normal. It is well known that the diagnostic efficacy of MRV without contrast medium is lower than that of MRV with contrast medium. However, since the main objective of our study was to determine variations, MRV without contrast medium is also sufficient according to these results. Furthermore, considering the possible side effects associated with unnecessary contrast medium use, the use of contrast-free MRV does not constitute a limitation for this study.

In the literature, there are many studies examining the AV of cerebral venous structures using MRV in pediatric cases [2, 3, 7]; however, only one study examines the relationships between the presence of AV and the development and complications of CSVT, and it has a limited population size [4]. The findings of that study regarding variation are similar to our results. As seen in both the literature and our study, AV prevalence has been shown to be comparable in patients with and without CSVT.

Cerebral venous variations are common in children, the most common being transverse sinus asymmetry, usually characterized by a hypoplastic or aplastic transverse sinus, and typically observed on the left side. Studies show that approximately 20–25% of pediatric individuals have an underdeveloped left transverse sinus, with the right side usually being dominant, and complete bilateral symmetry found in only about 10% of cases. Other variations, in order of frequency, include persistent occipital sinus due to persistent fetal canals, persistent falx sinus (midline vein running along the falx cerebri), superior sagittal sinus variations, and duplications or fenestrations of the dural sinuses. All of these anatomical differences are benign; however, it is important to know their frequency of occurrence to avoid confusion with sinus thrombosis in pediatric neuroimaging [7]. The distribution frequencies of anatomical variations (AV) detected in our study are consistent with the literature. The most frequent subgroups are hypoplasia/aplasia, followed by persistent fetal structures and then duplication/fenestration/septation.

The predominant anatomical difference identified in both the prior work investigating the correlation between AV and CSVT [4] and our cohort is the left hypoplastic transverse sinus. In the aforementioned study, the prevalence of hypoplastic/aplastic sinus was almost 80%, but it was observed at a reduced frequency of about 50% in our sample. The prevalence of permanent fetal structures in our sample exceeded that reported by Kouzmitcheva et al. [4], although the incidence of the disconnected superficial and deep systems variants was comparable. Moreover, while assessing the prevalence of duplication, fenestration, and septation changes across both groups, our research identified a reduced incidence in CSVT cases, although the rates in the normal group were comparable in both investigations [4]. The distinctions noted in AV subtypes are fundamentally unique factors that represent population characteristics. Furthermore, although a broader spectrum of AVs was identified in the previously mentioned [4] article, the diversity of AVs within our study population was comparatively limited. Our study data indicates that duplication, fenestration, and septation variations were more common in the control group, whereas variations in disconnected superficial and deep systems were more frequently observed in the CSVT group. However, the limited sample size of patients hindered a statistically significant analysis. The aforementioned circumstances may be regarded as somewhat restrictive constraints.

The most common complications in patients with CSVT include acute epileptic seizures, hemorrhagic infarction, permanent neurological sequelae, increased intracranial pressure, and death. According to recent studies, the overall mortality rate of CSVT in children is approximately 4–6%; however, the risk of developing complications can increase mortality, especially in the presence of certain risk factors. Newborns account for approximately one-third of CSVT cases, and significantly higher complication and mortality rates have been reported in the literature compared to other children. However, most older children tend to recover completely [8, 9].

Similar to the literature, papilledema, elevated intracranial pressure, and cerebral venous infarction were detected in our study population. As expected, most of the CSVT group recovered without complications, and the median age of those with complications was found to be statistically significantly lower. There are no similar studies in the literature examining the relationship between the presence of AV and the development of complications. Only Kouzmitcheva et al.‘s [4] study examined the relationship between the presence of increased intracranial pressure and the detection of AV, emphasizing a lack of a significant relationship. As a broader contribution to the literature, our data also suggest that AV detection rates are similar in cases with and without complications.

To summarize the strengths of our study, it represents the largest population study in the literature examining the presence of AV and the detection of CSVT. Furthermore, it is the only study to examine the relationship between the presence of AV and the development of complications. Furthermore, the age and gender balance between the CSVT and control groups, and the broad consistency of symptomatology, complication types, and age-related demographic data in the CSVT cases with the literature, are considered factors that enhance the reliability of the study.

Our study also has some limitations. Being a single-center retrospective study is the most significant limitation. As can be said for all retrospective studies, expanding the population would increase data reliability. The relatively small number of cases using IVCM can be considered another limitation; however, its relatively minor nature was detailed in the discussion. As a non-interventional characteristic of the population, not every AV could be identified and included in the study. There are some variations reported in the literature but not observed in our population. Although a trend toward an association between anatomical variations and CSVT was observed, the limited sample size may have reduced statistical power and prevented the detection of statistically significant differences.

In conclusion, AVs are often identified during MRI scans. CSVT is a significant contributor to stroke, resulting in death and morbidity among the pediatric population. The association between the occurrence of AV and CSVT and/or complications linked to CSVT has not been investigated in extensive populations. Our study data indicates that the existence of AV does not influence CSVT or complications associated with CSVT.

## Data Availability

The data are contained within the article and/or supplementary material.

## References

[1] Otite FO, Vanguru H, Anikpezie N, Patel SD, Chaturvedi S. Contemporary incidence and burden of cerebral venous sinus thrombosis in children of the United States. Stroke. 2022;53(12):e496–9. 10.1161/STROKEAHA.122.039822 36321458

[2] Sarma A, Martin D, Pruthi S, Jones R, Little SB. Imaging the cerebral veins in pediatric patients: beyond dural venous sinus thrombosis. Radiographics. 2023;43(2):e220129. 10.1148/rg.220129 36656758

[3] Goyal G, Singh R, Bansal N, Paliwal VK. Anatomical variations of cerebral MR venography: is gender matter? Neurointervention. 2016;11(2):92–98. 27621945 10.5469/neuroint.2016.11.2.92 PMC5018554

[4] Kouzmitcheva E, Andrade A, Muthusami P, Shroff M, MacGregor DL, Deveber G, et al. Anatomical venous variants in children with cerebral sinovenous thrombosis. Stroke. 2019;50(1):178–80. 30580715 10.1161/STROKEAHA.118.023482

[5] Ichord R. Cerebral sinovenous thrombosis. Front Pediatr. 2017;5:163. 28798906 10.3389/fped.2017.00163 PMC5529336

[6] Saposnik G, Bushnell C, Coutinho JM, Field TS, Furie KL, Galadanci N, et al. Diagnosis and management of cerebral venous thrombosis: a scientific statement from the American Heart Association. Stroke. 2024;55(3):e77–90. 10.1161/STR.0000000000000456 38284265

[7] Zedde M, Pascarella R. Non-thrombotic filling defects in cerebral veins and sinuses: when normal structures mimic a disease. Neurol Int. 2025;17(1):9. 39852773 10.3390/neurolint17010009 PMC11767902

[8] Proaño JS, Martinez PA, Sendi P, Totapally BR. Characteristics and outcomes of children with cerebral sinus venous thrombosis. Neurocrit Care. 2023;39(2):331–38. 37438549 10.1007/s12028-023-01765-7

[9] Cornelius LP, Elango N, Jeyaram VK. Clinico-etiological factors, neuroimaging characteristics and outcome in pediatric cerebral venous sinus thrombosis. Ann Indian Acad Neurol. 2021;24(6):901–07. 35359540 10.4103/aian.AIAN_221_21 PMC8965941

